# Effect of Urate-Lowering Therapy on the Progression of Kidney Function in Patients With Asymptomatic Hyperuricemia: A Systematic Review and Meta-Analysis

**DOI:** 10.3389/fphar.2021.795082

**Published:** 2022-01-18

**Authors:** Lin Zhang, Kang An, Xingyu Mou, Mei Zhang, Qiaoli Su, Shuangqing Li

**Affiliations:** ^1^ Department of General Practice, West China Hospital, Sichuan University, Chengdu, China; ^2^ Department of Laboratory Medicine, West China Hospital, Sichuan University, Chengdu, China

**Keywords:** urate-lowering therapy, asymptomatic hyperuricemia, eGFR slopes, kidney events, chronic kidney disease

## Abstract

**Background:** Hyperuricemia is involved in the risk of chronic kidney disease (CKD). However, whether urate-lowering therapy (ULT) can influence the progression of kidney function in patients with asymptomatic hyperuricemia is still controversial. We conducted a systematic review and meta-analysis to evaluate the effect of ULT on the progression of kidney function in asymptomatic hyperuricemia patients.

**Methods:** The MEDLINE, EMBASE and Cochrane databases were searched without language, national or ethnic restrictions for randomized controlled trials published prior to November 30, 2020, that compared ULT with controlled therapy in patients with asymptomatic hyperuricemia.

**Results:** Eleven studies were included for qualitative synthesis. ULT did not ameliorate eGFR slopes (WMD 0.36 ml/min/1.73 m^2^ per year, 95% CI: −0.31, 1.04), or lead to reductions in kidney events (RR 1.26; 95% CI: 0.80, 2.00) or all-cause mortality (RR 1.00; 95% CI: 0.65, 1.55), although ULT resulted in a decrease in serum uric acid levels (WMD −2.73 mg/dl; 95% CI: −3.18, −2.28) and lowered the incidence of gout episodes (0.9 vs 2.7%, RR 0.38; 95% CI: 0.17, 0.86).

**Conclusion:** In patients with asymptomatic hyperuricemia, ULT did not decay the progression of kidney function. Long-term and larger sample studies are needed to verify the results.

**Systematic Review Registration**: [www.crd.york.ac.uk/PROSPERO/#recordDetails], identifier [CRD42020204482].

## Introduction

Hyperuricemia is often accompanied by traditional metabolic abnormalities, such as cardiovascular diseases, type 2 diabetes, hypertension and obesity, and was shown to be an independent risk factor for all-cause and cardiovascular mortality with a mild elevation of serum uric acid levels in a nationwide community-based population followed up for 7 years (Konta et al., 2020). Chronic kidney disease (CKD) is defined as the presence of structural or functional abnormalities of the kidney lasting for more than 3 months. Cardiovascular disease, end-stage renal disease requiring renal replacement therapy (RRT), and mortality increase with a decrease in the glomerular filtration rate (GFR) (Gansevoort et al., 2013). It is essential to detect and mitigate the possible risk factors for kidney function deterioration. Several studies have shown that uric acid is an independent risk factor for the occurrence and progression of kidney diseases (Bellomo et al., 2010; Li et al., 2014; Oh et al., 2019; Zhou et al., 2019) and is associated with a significant decrease in kidney function (Tsai et al., 2017). Hyperuricemia is involved in the risk of incident RRT and all-cause mortality in patients with stage 3–5 CKD (Lee and Wang, 2019). However, correlational relationships do not represent causation.

Asymptomatic hyperuricemia and gout are continuous pathological processes of hyperuricemia. Performing interventions to treat gout in patients with CKD can have benefits such as delaying and ameliorating renal function(Novella-Navarro et al., 2020). This issue has been affirmed and recommended by guidelines in China and America. However, whether asymptomatic hyperuricemia should be treated in patients with CKD remains controversial. A randomized controlled trial (RCT) with a follow-up of 6 months showed that febuxostat had the ability to delay decreases in the GFR in patients with asymptomatic hyperuricemia and CKD by decreasing serum uric acid levels (Sircar et al., 2015). Nevertheless, an RCT with a duration of 108 weeks of follow-up demonstrated that febuxostat did not delay the advancement of kidney function in patients with similar conditions (Kimura et al., 2018). The guidelines of America did not recommend treatment for these patients (Fitzgerald et al., 2020). In China’s guidelines, it is suggested that patients with asymptomatic hyperuricemia should be treated with urate-lowering drugs when the level of serum uric acid ≥540 μmol/L or 480 μmol/L with one of the following comorbidities: hypertension, abnormal lipid metabolism, diabetes, obesity, stroke, coronary heart disease, cardiac insufficiency, urinary acid nephrosis, or renal function damage (≥CKD stage 2) (Chinese Society of Endocrinology, 2020). A Cochrane systematic analysis showed that in patients with or without CKD, ULT may prevent the development of CKD (Sampson et al., 2017). Su et al. (Su et al., 2017) performed a meta-analysis and demonstrated that ULT can reduce the relative risk (95% CI, 31, 64) of kidney failure events by 55% in patients with CKD. Unfortunately, these studies did not distinguish between patients with gout and those with asymptomatic hyperuricemia, which may muddle the interpretation of the results.

We performed a systematic review and meta-analysis to evaluate the efficacy of urate-lowering therapy on the progression of kidney function in patients with asymptomatic hyperuricemia.

## Methods

### Protocol

We carried out the systematic review and meta-analysis in accordance with the Preferred Reporting Items for Systematic Reviews and Meta-Analyses (PRISMA) statement. The protocol of our study was registered in PROSPERO (No. CRD42020204482).

### Search Strategies and Selection Criteria

The MEDLINE, Embase and Cochrane databases were searched until November 30, 2020, without limitations on language, country, or race.

The following terms and their relevant formations were used: “xanthine oxidase inhibitor OR allopurinol OR febuxostat OR topiroxostat OR pegloticase OR probenecid OR puricase OR urate lowering therapy” AND “asymptomatic hyperuricemia” OR “hyperuricemia” AND “randomized controlled trial”.

### Inclusion and Exclusion Criteria

Studies meeting the following criteria were included: 1) patients aged more than 18 years old with asymptomatic hyperuricemia (serum uric acid concentration ≥6.8 mg/dl (Fitzgerald et al., 2020) or uric acid level of women ≥360 umol/L), if the mean serum uric acid level ≥6.8 mg/dl in all included patients, the study was included; 2) patients have had subcutaneous tophi or have had gout, but no acute episodes in the past 1 year; 3) randomized controlled trials lasted more than 3 months and compared urate-lowering therapy with placebo or traditional treatment. The exclusion criteria were as follows: 1) patients currently experiencing gout; 2) trials compared the efficacy of different urate-lowering therapies or different doses of the same drugs.

### Outcome Measures of Efficacy and Safety

The primary outcome of efficacy was the change of eGFR slopes. The secondary endpoints were changes in estimated GFR (eGFR), kidney events (kidney failure (defined as GFR< 15 ml/min/1.73 m^2^) or end stage kidney disease (ESKD) (defined as treatment with maintenance dialysis or kidney transplantation) or established surrogate end point: doubling of serum creatinine (equivalent to 57% decline in eGFRcr) or more than 40% estimated GFR decline) (Levey et al., 2014; Kanda et al., 2018), uric acid levels, and blood pressure, gout episodes and all-cause mortality.

### Data Extraction and Risk of Bias

Based on the inclusion and exclusion criteria, two researchers (XM and KA) independently screened the full text of the eligible studies and carried out data extraction. Baseline patient demographics, interventions, outcomes and adverse events were extracted. Any inconsistencies between them were resolved by further discussion in consultation with a third reviewer if necessary (LZ).

MZ and QS used the Cochrane Collaboration’s risk of bias tool to conduct the methodological quality assessment shown as risk of bias of the included RCTs (Higgins et al., 2011), including assessments of random sequence generation, allocation concealment, blinding of outcome assessors, selective outcome reporting, and other items. Studies were rated as having either a low, high, or unclear risk of bias. Any disagreements were resolved by discussion and consultation with a third reviewer (S.Q.L.).

### Data Synthesis and Analysis

Data synthesis was conducted with RevMan (version 5.2). Weighted mean differences (WMDs), 95% confidence intervals (CIs) for continuous effects and risk ratios (RRs) for dichotomous effects were calculated. If more than two arms were included in one intervention in one study, the effect sizes were combined to obtain a mean difference. When the standard deviation (SD) of continuous data changes was not reported in the study, it was calculated according to the equation provided in the Cochrane System Review Handbook. The I^2^ value was used to assess the heterogeneity between the included studies; low, moderate and high heterogeneity were indicated when the I^2^ value was less than 25%, 25%–50% and more than 50%, respectively. A random effects model was selected regardless of the I^2^ value for statistical analysis. Begg’s funnel graph was used to evaluate the potential publication bias by Stata (version 14, StataCorp. 2015) due to the subjectivity of inspecting the symmetry, Egger’s test was also performed to assess the bias. Publication bias may exist when Begg’s funnel diagram is considered asymmetrical and when Egger’s test has a *p* value less than 0.05. Subgroup analysis for changes in eGFR was employed based on the number of participants (“less than or equal to” or more than 100 patients in each group). Sensitivity analysis was employed to appraise the stability of the pooled WMD of uric acid levels.

## Results

### Search Results

**FIGURE 1:**
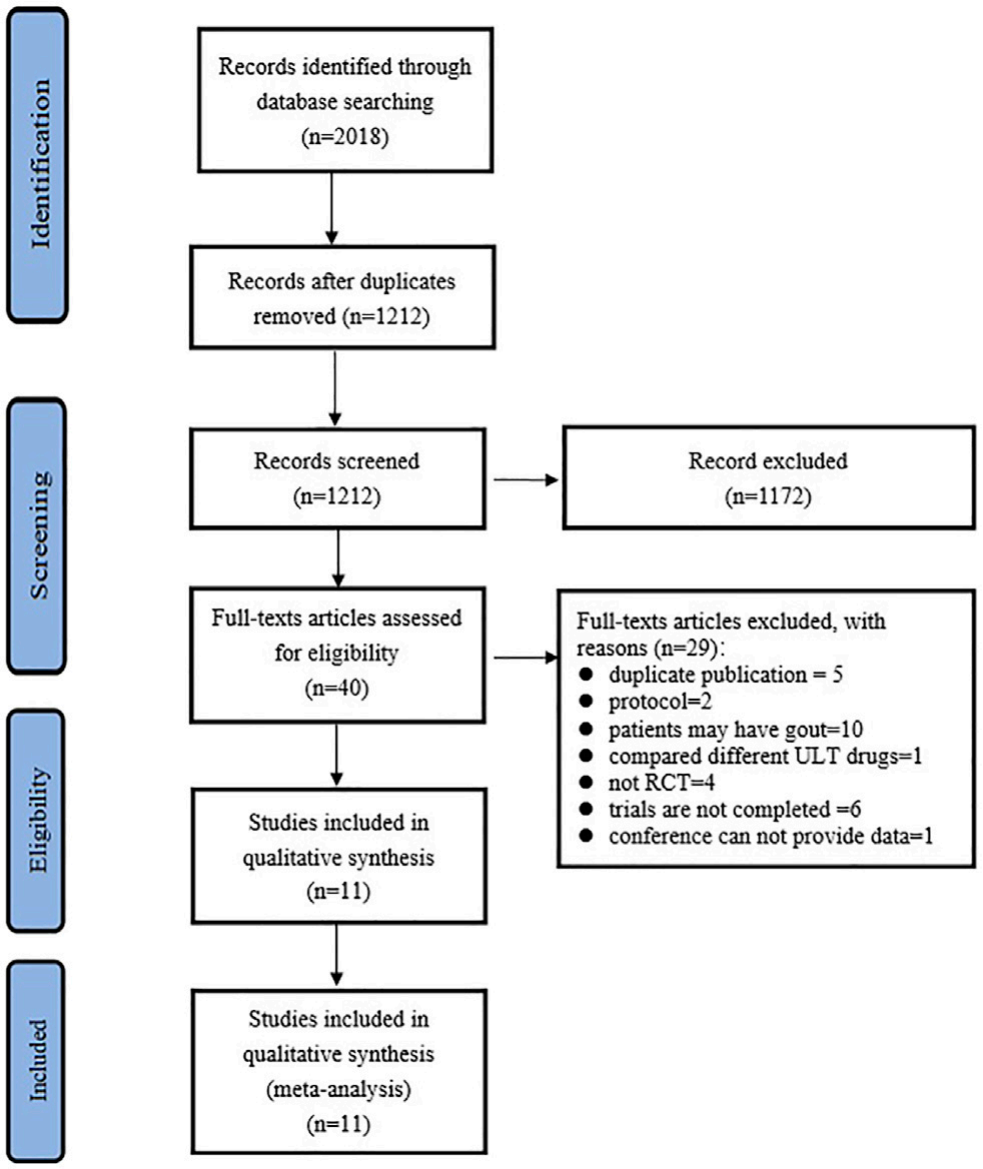
PRISMA Flow diagram of the study.

The PRISMA selection flow chart is shown in Figure 1. Through the database searching, a total of 2,018 records were identified. A total of 1212 records were identified after duplicate removal, and 40 full-text articles were assessed for eligibility. Finally, 11 studies (Siu et al., 2006; Liu et al., 2015; Sircar et al., 2015; Takir et al., 2015; Golmohammadi et al., 2017; Jalal et al., 2017; Kimura et al., 2018; Mukri et al., 2018; Kojima et al., 2019; Badve et al., 2020; Tanaka et al., 2020) were included for qualitative synthesis.

### Clinical Characteristics

The patients’ baseline demographics, characteristics, intervention arms, and study duration are exhibited in Supplementary Table S1. A total of 1,551 and 1,544 subjects were included in the uric-lowering therapy group and control group, respectively. The duration of the studies was between 12 weeks and 3 years. The uric-lowering drugs were allopurinol (Siu et al., 2006; Liu et al., 2015; Takir et al., 2015; Golmohammadi et al., 2017; Jalal et al., 2017; Badve et al., 2020) and febuxostat (Sircar et al., 2015; Kimura et al., 2018; Mukri et al., 2018; Kojima et al., 2019; Tanaka et al., 2020). The included trials were either multicenter trials (Jalal et al., 2017; Kimura et al., 2018; Kojima et al., 2019; Badve et al., 2020; Tanaka et al., 2020), or single-center trials (Siu et al., 2006; Liu et al., 2015; Sircar et al., 2015; Takir et al., 2015; Golmohammadi et al., 2017; Mukri et al., 2018). Serum uric acid levels ranged from 7.26 ± 0.15 to 9.92 ± 1.68 mg/dl, and the eGFR ranged from 20.84 ± 5.80 to 90.1 ± 18.4 ml/min/1.73 m^2^. The eGFR in the included patients was less than 60 ml/min/1.73 m^2^ in eight studies (Sircar et al., 2015; Golmohammadi et al., 2017; Jalal et al., 2017; Kimura et al., 2018; Mukri et al., 2018; Kojima et al., 2019; Badve et al., 2020; Tanaka et al., 2020), more than 60 ml/min/1.73 m^2^ in one study (Liu et al., 2015), and not mentioned in detail in two studies (Siu et al., 2006; Takir et al., 2015).

### Risk of Bias of Included Studies

The risk of bias of the included studies was estimated by the Cochrane Collaboration’s risk of bias tool (Supplementary Figure S1). Nine studies (Siu et al., 2006; Liu et al., 2015; Sircar et al., 2015; Jalal et al., 2017; Kimura et al., 2018; Mukri et al., 2018; Kojima et al., 2019; Badve et al., 2020; Tanaka et al., 2020) described random sequence generation clearly. Blinding of participants and personnel (performance bias) was mentioned in three studies (Sircar et al., 2015; Jalal et al., 2017; Badve et al., 2020), unclear in one study (Golmohammadi et al., 2017); hence seven studies (Siu et al., 2006; Liu et al., 2015; Takir et al., 2015; Kimura et al., 2018; Mukri et al., 2018; Kojima et al., 2019; Tanaka et al., 2020) had a high risk of bias for this aspect. Allocation concealment was unclear in six studies (Siu et al., 2006; Liu et al., 2015; Takir et al., 2015; Golmohammadi et al., 2017; Kimura et al., 2018; Mukri et al., 2018).

### eGFR Slopes

**FIGURE 2:**
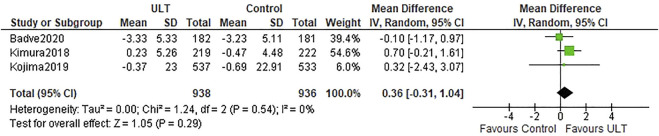
eGFR slopes: ULT vs control group.

Three studies (Kimura et al., 2018; Kojima et al., 2019; Badve et al., 2020) described the eGFR slopes. There was no difference in the eGFR slopes between the ULT group and the control group (WMD 0.36 ml/min/1.73 m^2^ per year; 95% CI: −0.31, 1.04) (Figure 2).

### eGFR

Seven studies (Liu et al., 2015; Sircar et al., 2015; Golmohammadi et al., 2017; Jalal et al., 2017; Kimura et al., 2018; Mukri et al., 2018; Tanaka et al., 2020) presented the results or changes in eGFR before and after interventions. The results were presented as median (IQR) in one study (Mukri et al., 2018). We could not obtain the mean and standard deviation, hence the study was not included in our statistical analysis. The pooled results showed that ULT could improve renal function compared with control groups (WMD 2.93 ml/min/1.73 m^2^; 95% CI: 1.00, 4.87) with obvious heterogeneity (I^2^ = 80%) (Supplementary Figure S2).

However, the other studies did not provide the change of eGFR, especially in the study of Kojima et al., which included a large number of subjects and did not show the difference between febuxostat and control groups. These may lead to the bias of evaluating the effect of ULT on eGFR. When the studies with the number of participants≤100 in each group were excluded, ULT did not ameliorate eGFR (WMD 0.56 ml/min/1.73 m^2^; 95% CI: −0.42, 1.53) with no heterogeneity (I^2^ = 0%) (Supplementary Figure S3).

### Kidney Events

**FIGURE 3:**
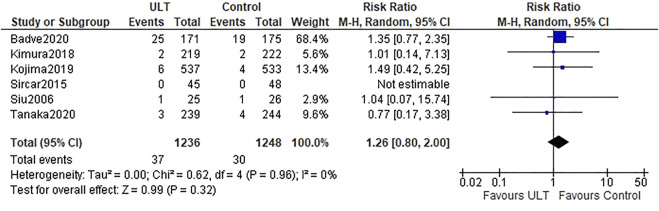
Kidney events: ULT vs control group.

Six studies (Siu et al., 2006; Sircar et al., 2015; Kimura et al., 2018; Kojima et al., 2019; Badve et al., 2020; Tanaka et al., 2020) referred to the kidney events. Overall, there were 37 (37 of 1,236 patients, 3.0%) and 30 (30 of 1,248 patients, 2.4%) kidney events in the ULT and control groups, respectively. There was no difference between the ULT and control groups (RR 1.26; 95% CI: 0.80, 2.00) (Figure 3).

### Uric Acid

**FIGURE 4:**
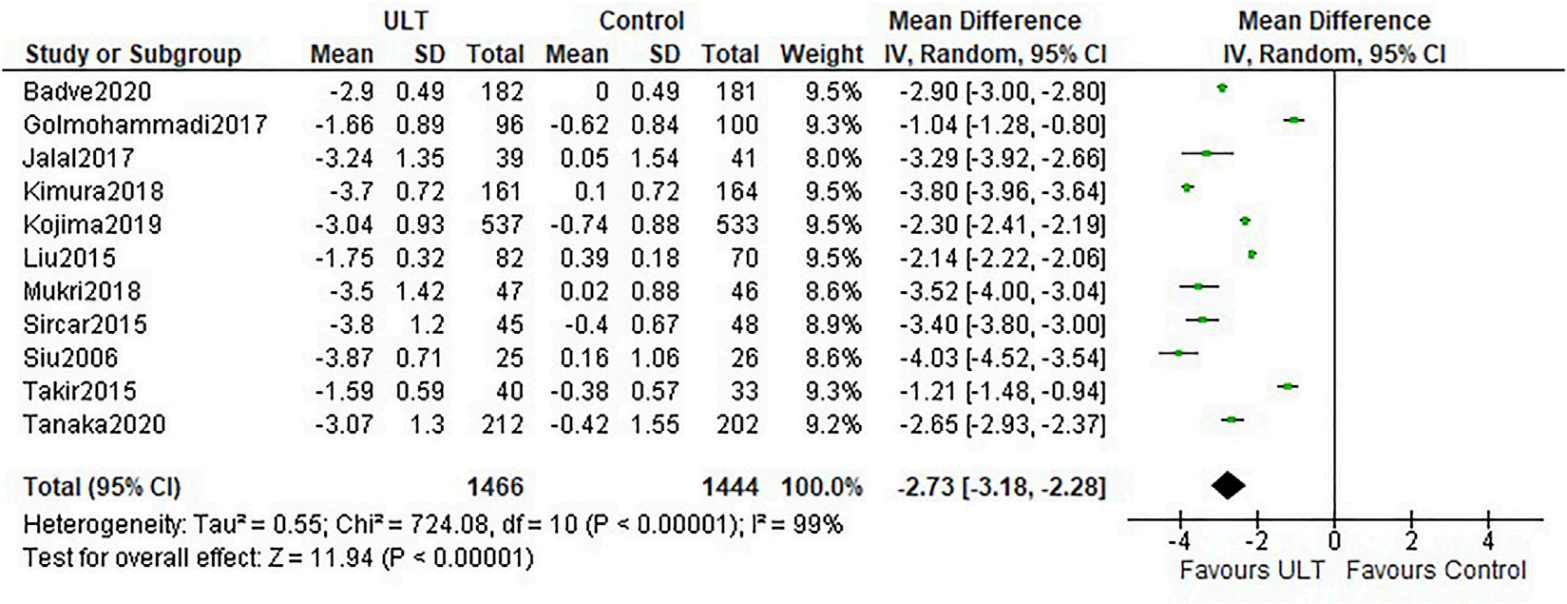
Uric acid levels: ULT vs control group.

All the studies reported uric acid levels before and after intervention. Compared with the control group, ULT significantly lowered serum uric acid levels (WMD -2.73 mg/dl; 95% CI: −3.18, −2.28) (Figure 4). Both allopurinol and febuxostat notably decreased serum uric acid levels.

For the Begg’s plot (Supplementary Figure S4A) of uric acid, the studies were distributed approximately symmetrically. The *p* value for the Egger’s test was 0.095 (Supplementary Figure S4B) and the Egger’s plot shown in Supplementary Figure S4C, suggested a low risk of publication bias.

Sensitivity analysis was performed by excluding each study one by one; and the difference did not change, which showed the stability of the result.

### Blood Pressure

ULT had the ability to decrease systolic blood pressure (WMD −3.88 mmHg; 95% CI: −4.85, −2.91) (Supplementary Figure S5) and diastolic blood pressure (WMD −2.41 mmHg; 95% CI: −3.31, −1.52) (Supplementary Figure S6).

### Gout Episodes

Five studies (Jalal et al., 2017; Kimura et al., 2018; Kojima et al., 2019; Badve et al., 2020; Tanaka et al., 2020) reported the incidence of gout episodes. The incidence of gout episodes was 0.9% (11/1,216) and 2.7% (33/1,221) in the ULT and control groups, respectively. Urate-lowering treatment seemed to decrease the incidence of gout episodes when compared with the control (RR 0.38; 95% CI: 0.17, 0.86) (Supplementary Figure S7).

### All-Cause Mortality

Eight studies provided information on the incidence of all-cause mortality. Forty patients died in each of the groups i.e., ULT (3.1%) and control (3.0%). There was no difference in all-cause mortality between the ULT and control groups (RR 1.00; 95% CI: 0.65, 1.55) (Supplementary Figure S8).

## Discussion

Our meta-analysis evaluated the kidney outcomes of ULT in patients with asymptomatic hyperuricemia and found that compared with the control group, ULT did not ameliorate eGFR slopes, or lead to reduction in kidney events or all-cause mortality, although ULT resulted in a decrease in serum uric acid levels and lowered the incidence of gout episodes.

There is consensus on initiating ULT when a patient has gout. Nevertheless, this may not be the case in patients without gout or asymptomatic hyperuricemia. Our results demonstrated that ULT did not reduce eGFR slopes or kidney events in patients without gout. Although several theoretical mechanisms provided the possibility of the damaging effects of uric acid in causing CKD (Sanchez-Lozada et al., 2002; Sanchez-Lozada et al., 2008), our results did not support them.

Gout episodes are caused by the incorporation of crystalloid salts of uric acid into the joints. With the rise of uric acid levels, the risk of acute gout episodes increases simultaneously. Our results showed that ULT lowered the incidence of gout episodes in patients without gout. Nevertheless, the incidence of gout episodes events was 1.27 and 2.71% in the ULT and control groups respectively, which was relatively low.

The impact of uric acid on hypertension is of research interest. A single-center longitudinal cohort study with nearly 20 years of follow-up indicated that elevated serum uric acid was related to the progression of hypertension (Wang et al., 2014). A meta-analysis including 25 studies with 97,824 participants showed that hyperuricemia increased the risk of incident hypertension (Wang et al., 2014). Increased uric acid concentrations were associated with a high risk of the development of hypertension (Grayson et al., 2011). ULT treatment exhibited a reduction hazard ratio and risks of hypertension (Feig et al., 2008; Lin et al., 2020) and lowered blood pressure in patients with hyperuricemia and hypertension (Gunawardhana et al., 2017; Qu et al., 2017). Our results showed the possible benefit of ULT in subjects without gout.

Hyperuricemia has been shown to be an independent risk factor for mortality in patients with chronic obstructive pulmonary disease (Zhang et al., 2015). A retrospective case-matched cohort study revealed that patients with asymptomatic hyperuricemia had an increased risk of all-cause and CVD mortality and that ULT can reduce the risk of all-cause death (Chen et al., 2015). However, this was in contrast to our result, which indicated that there was no difference between the ULT and control groups in all-cause mortality. This finding needs to be investigated further.

### Limitations

There were some limitations in our review. First, the equations for evaluating eGFR were CKD-EPI (Jalal et al., 2017; Mukri et al., 2018; Badve et al., 2020), the Japanese eGFR equation (Kimura et al., 2018; Kojima et al., 2019), MDRD (Sircar et al., 2015) or were not reported (Siu et al., 2006; Liu et al., 2015; Takir et al., 2015; Golmohammadi et al., 2017; Tanaka et al., 2020), which may influence the assessment of the real GFR of included subjects. Second, the only included ULTs were allopurinol and febuxostat, which may lead to bias in assessing the effects of ULTs. Furthermore, since only three studies mentioned blinding of participants and personnel, specifically, therefore, the possibility of performance bias may exit. In addition, some studies did not provide the change of eGFR slopes and eGFR, which may lead to bias in evaluating the efficacy of ULT.

## Conclusion

In conclusion, our meta-analysis evaluated the effect of ULT on kidney function in patients with asymptomatic hyperuricemia. Compared with the control group, ULT did not ameliorate eGFR slopes, or lead to reductions in kidney events or all-cause mortality, although ULT resulted in a decrease in serum uric acid levels and lowered the incidence of gout episodes. Long-term and larger sample studies are needed to verify the kidney outcomes of ULT in patients with asymptomatic hyperuricemia.

## Data Availability

The original contributions presented in the study are included in the article/Supplementary Material, further inquiries can be directed to the corresponding author.
