# Supplementary material for: Effect of Urate-Lowering Therapy on the Progression of Kidney Function in Patients With Asymptomatic Hyperuricemia: A Systematic Review and Meta-Analysis
Source: Front Pharmacol. 2022 Jan 18;12:795082. doi: 10.3389/fphar.2021.795082 (PMC8804353; doi:10.3389/fphar.2021.795082)
Supplement: Supplementary file 1 [file DataSheet1.docx]

| Trial | Study duration | Study arms included  in meta-analysis | Number  (n) | Age  (years) | Uric acid  (mg/dl) | | Creatinine  (mg/dl) | | eGFR  (ml/min/1.73m^2^) | | | eGFR formula |  |
| --- | --- | --- | --- | --- | --- | --- | --- | --- | --- | --- | --- | --- | --- |
| Kimura  2018 | 108weeks | Febuxostat | 219 | 65.3±11.8 | 7.8 ± 0.9 | | 1.25±0.26 | | 45.2 ± 9.5 | | | JSN-CKDI | |
|  |  | Placebo | 222 | 65.4±12.3 | 7.8 ± 0.9 | | 1.25±0.26 | | 45.2 ± 9.5 | | |  |  |
| Kojima  2019 | 36 months | Febuxostat | 537 | 75.4 ± 6.7 | 7.54 ±1.06 | |  | | 54.62 ±14.11 | | Japanese GFR equation | |  |
|  |  | Non-febuxostat* | 533 | 76.0 ± 6.5 | 7.50 ±1.03 | |  | | 55.35 ±15.16 | |  |  |  |
| Badve2020 | 104weeks | Allopurinol | 182 | 62.3±12.6 | 8.2±1.8 | |  | | 31.6±11.7 | | CKD-EPI | |  |
|  |  | Placebo | 181 | 62.6±12.9 | 8.2±1.7 | |  | | 31.9±12.4 | |  |  |  |
| Siu2006 | 12 months | Allopurinol | 25 | 47.7±12.9 | 9.75 ±1.18 | | 1.64±0.63 | |  | |  | |  |
|  |  | Standard therapy^$^ | 26 | 48.8±16.8 | 9.92±1.68 | | 1.86±0.69 | |  | |  | |  |
| Sircar2015 | 6 months | Febuxostat | 45 | 56.22±10.87 | 9.0±2.0 | | 2.21±0.75 | | 31.5±13.6 | | MDRD | |  |
|  |  | Placebo | 48 | 58.42±14.52 | 8.2±1.1 | | 2.22±0.63 | | 32.6±11.6 | |  |  |  |
| Tanaka  2020 | 24months | Febuxostat | 239 | 69.1 ±10.1 | 7.76 ± 0.98 | |  | | 56.26 ±15.41 | |  | |  |
|  |  | Standard therapy^$^ | 244 | 69.1 ±10.7 | 7.73 ±1.04 | |  | | 57.12 ±15.83 | |  | |  |
| Golmohammadi2017 | 12 months |  |  |  | mild GFR | severe GFR | mild GFR | severe GFR | mild GFR | severe GFR |  | |  |
|  |  | Allopurinol | 96 |  | 7.86±1.36 | 7.85±1.41 | 1.66 ± 0.30 | 3.40 ± 0.95 | 50.37±11.26 | 20.84 ± 5.80 |  | |  |
|  |  | placebo | 100 |  | 7.77±1.26 | 7.70±0.95 | 1.68 ± 0.37 | 2.89 ± 0.56 | 50.38 ± 13.22 | 24.57 ± 3.97 |  | |  |
| Jalal2017 | 12weeks | Allopurinol | 39 | 55.9±13.7 | 8.3±1.4 | | 1.81±0.37 | | 41.3±8.9 | | CKD-EPI | |  |
|  |  | placebo | 41 | 58.9±9.3 | 8.7±1.6 | | 1.75±0.42 | | 42.4±9.6 | |  | |  |
| Liu2015 | three years | Allopurinol | 82 | 50 ± 10 | 433 ± 11& | | 75.6 ± 11.0^&^ | | 90.1 ± 17.5 | |  | |  |
|  |  | Conventional  treatment group^⁑^ | 70 | 51 ± 11 | 432 ± 9& | | 75.0 ± 10.9^&^ | | 90.1 ± 18.4 | |  |  |  |
| Mukri2018 | 6  months | Febuxostat | 47 | 64 (10)^#^ | 539.5 ± 104^&^ | | 195.2 ± 51.8^&^ | | 26.2 (14.3)^#^ | | CKD-EPI | |  |
|  |  | No treatment | 46 | 67 (6)^#^ | 537.3 ± 70.6^&^ | | 194.2 ± 56.2^&^ | | 28.2 (19.8)^#^ | |  |  |  |
| Takir2015 | 3 months | Allopurinol | 40 | 52.15± 15.86 | 7.86 ± 0.62 | | 0.9 ± 0.15 | |  | |  | |  |
|  |  | Control group^∆^ | 33 | 49.88 ± 12.46 | 7.45 ± 0.9 | | 1.07 ± 0.2 | |  | |  |  |  |

Table S1. Baseline characteristics of the study population

* Non-febuxostat:100 mg of oral allopurinol was considered if serum uric acid levels were elevated during the study period starting from the time of enrollment; $ Standard therapy: continue and adjust the usual therapy such as antihypertensive drugs, lipid-lowering agents, antiplatelet drugs or steroid or cytotoxic drugs, or non-pharmacological lifestyle modification for hyperuricemia, such as a healthy diet and exercise therapy; ∆Control group: non-pharmacological treatment of hyperuricemia. ⁑Conventional treatment group: received no uric acid lowering therapy. JSN-CKDI: Japanese Society of Nephrology-Chronic Kidney Disease Initiatives. & The unit is umol/L. Data are mean ±SD expect # (Data are median (IQR));

A


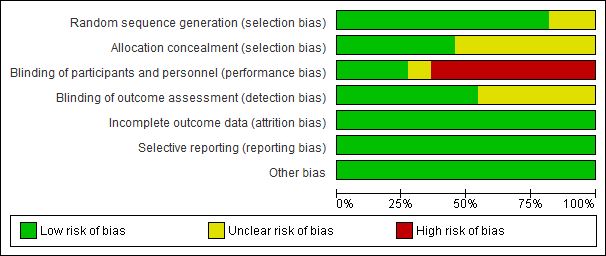


B


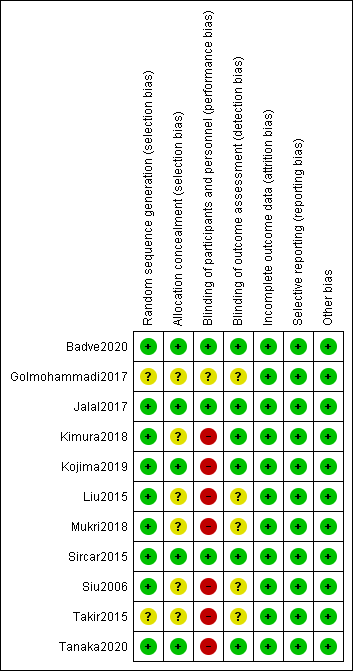


Supplemental Figure S1. Risk of bias of included studies (A) Risk of bias graph (B) Risk of bias summary


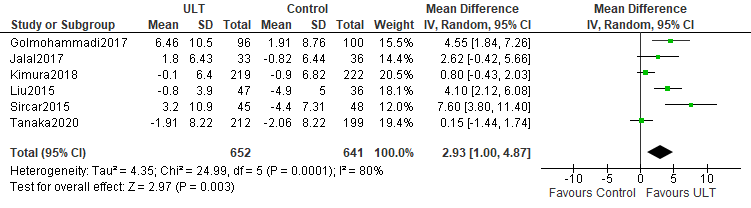


Supplemental Figure S2. eGFR: ULT *vs* control group


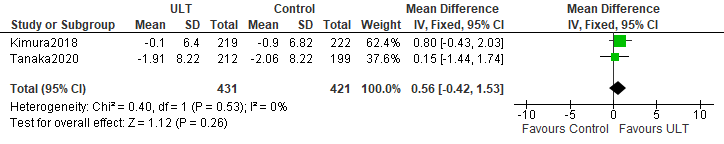


Supplemental Figure S3. eGFR of patients more than 100 in each group: ULT *vs* control group

A.

B. Egger's test

------------------------------------------------------------------------------

Std_Eff | Coef. Std. Err. t P>|t| [95% Conf. Interval]

-------------+----------------------------------------------------------------

slope | -1.540286 .695327 -2.22 0.054 -3.113225 .0326532

bias | -7.29893 3.909015 -1.87 0.095 -16.14174 1.543877

------------------------------------------------------------------------------

C.

Supplemental Figure S4. Publication bias (A) Begg's plot (B) P value for Egger's test (C) Egger's plot


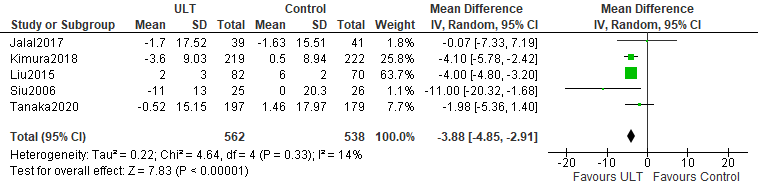


Supplemental Figure S5. Systolic blood pressure: ULT *vs* control group


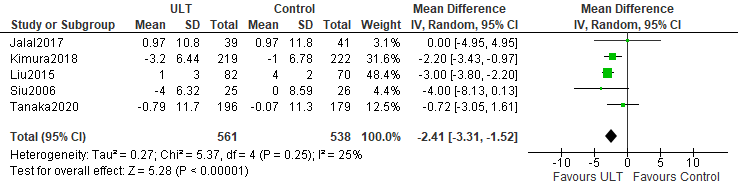


Supplemental Figure S6. Diastolic blood pressure: ULT *vs* control group


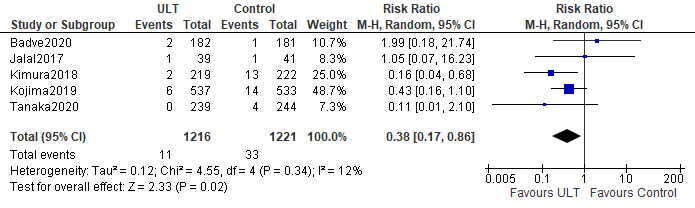


Supplemental Figure S7. Gout episodes: ULT *vs* control group


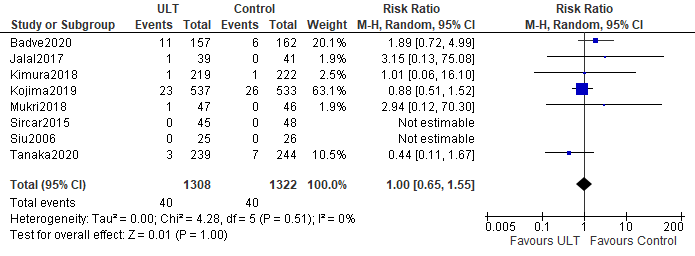


Supplemental Figure S8. All-cause mortality: ULT *vs* control group
